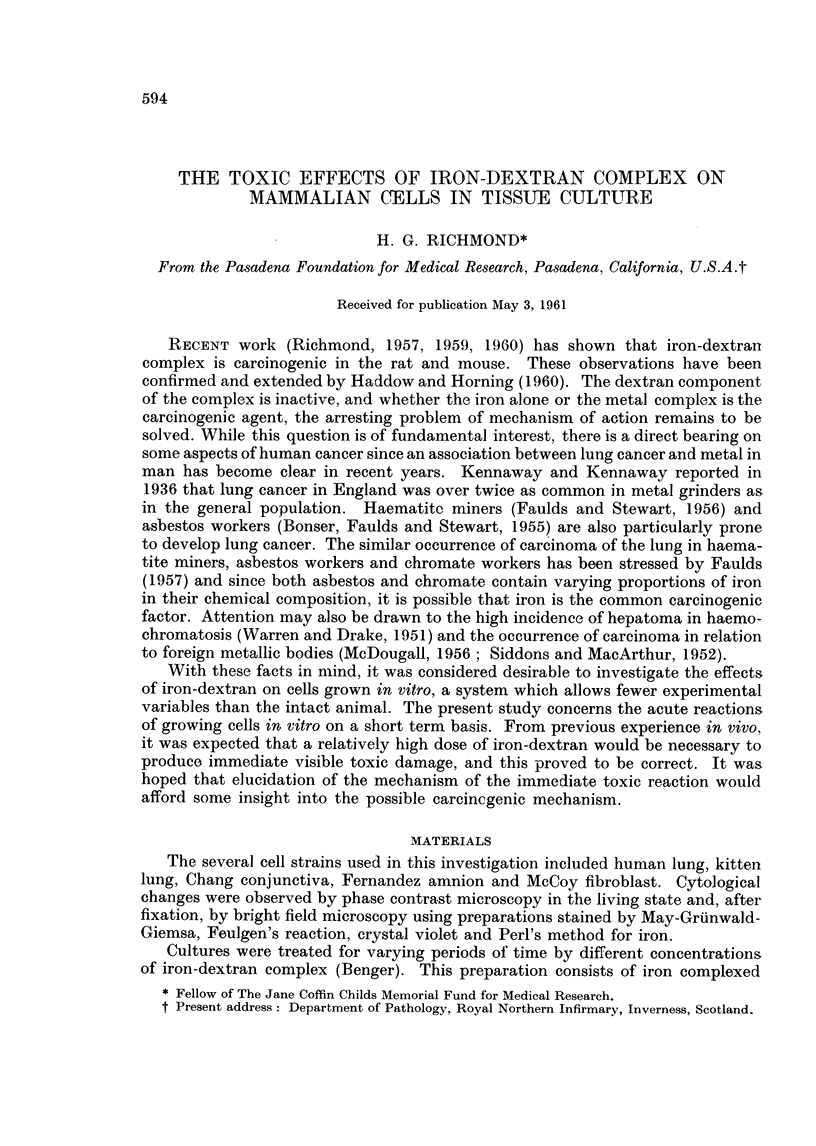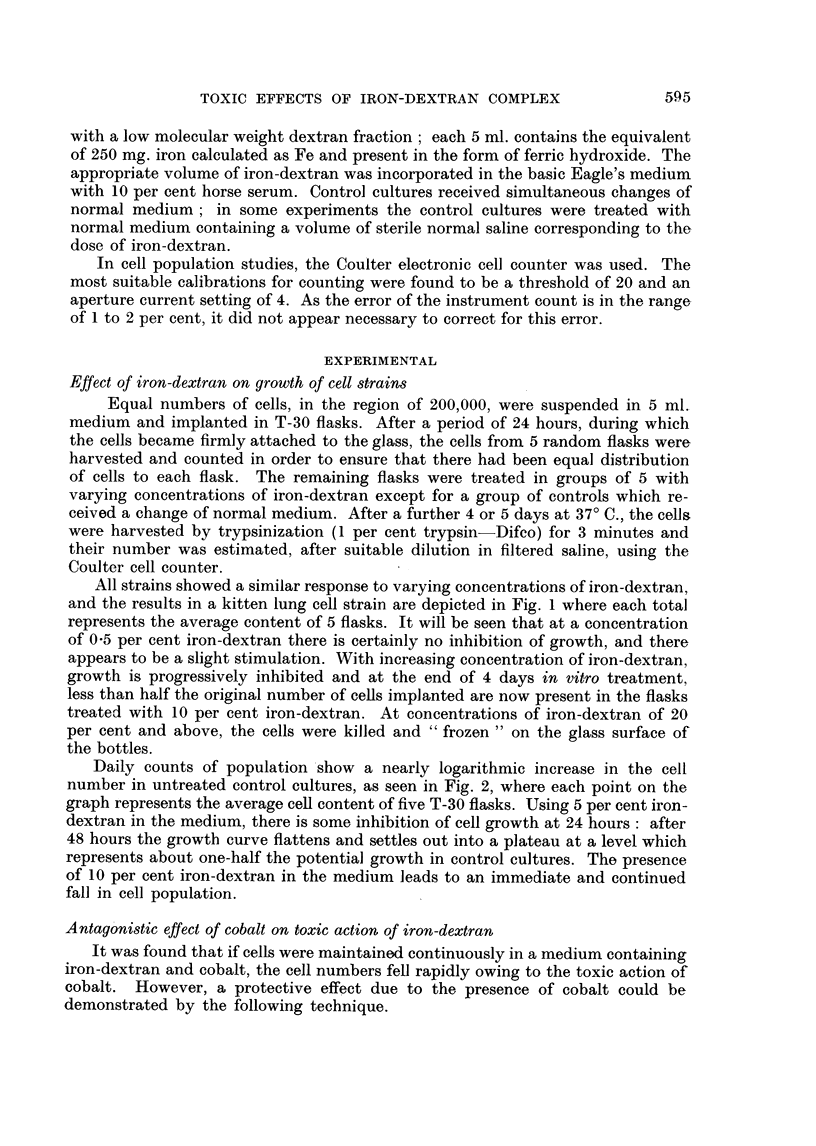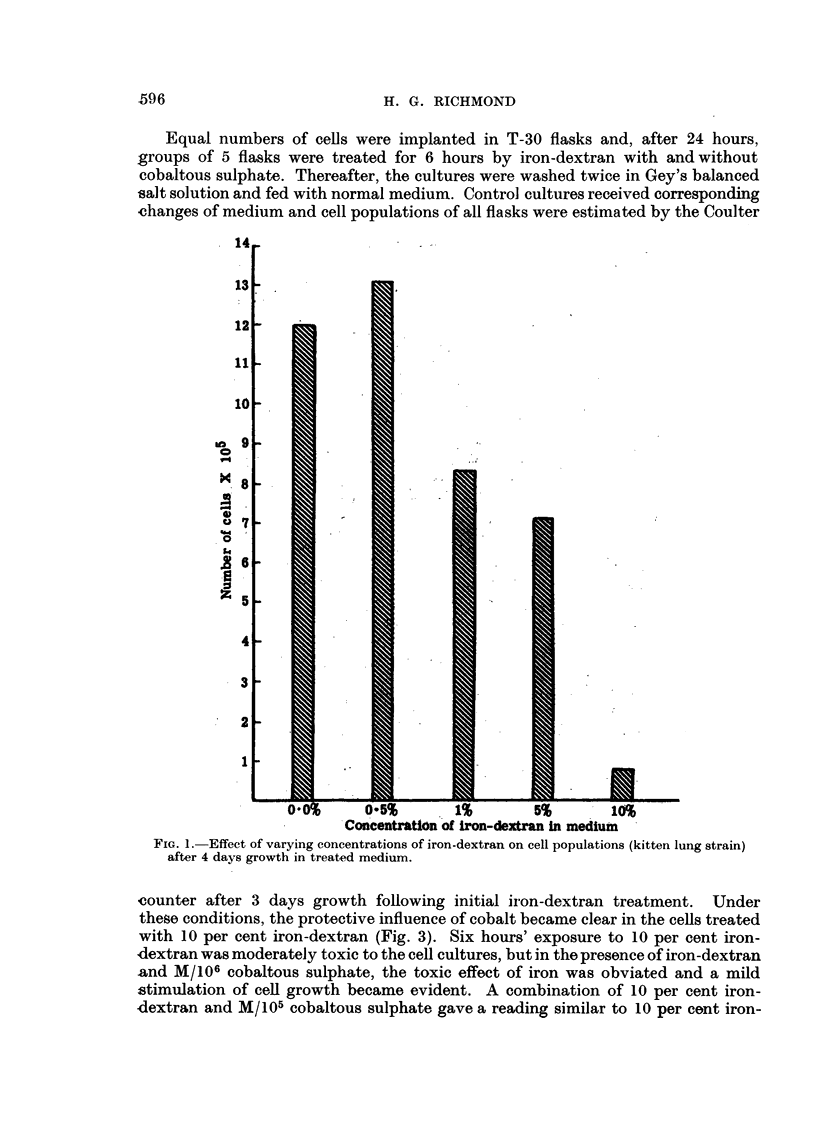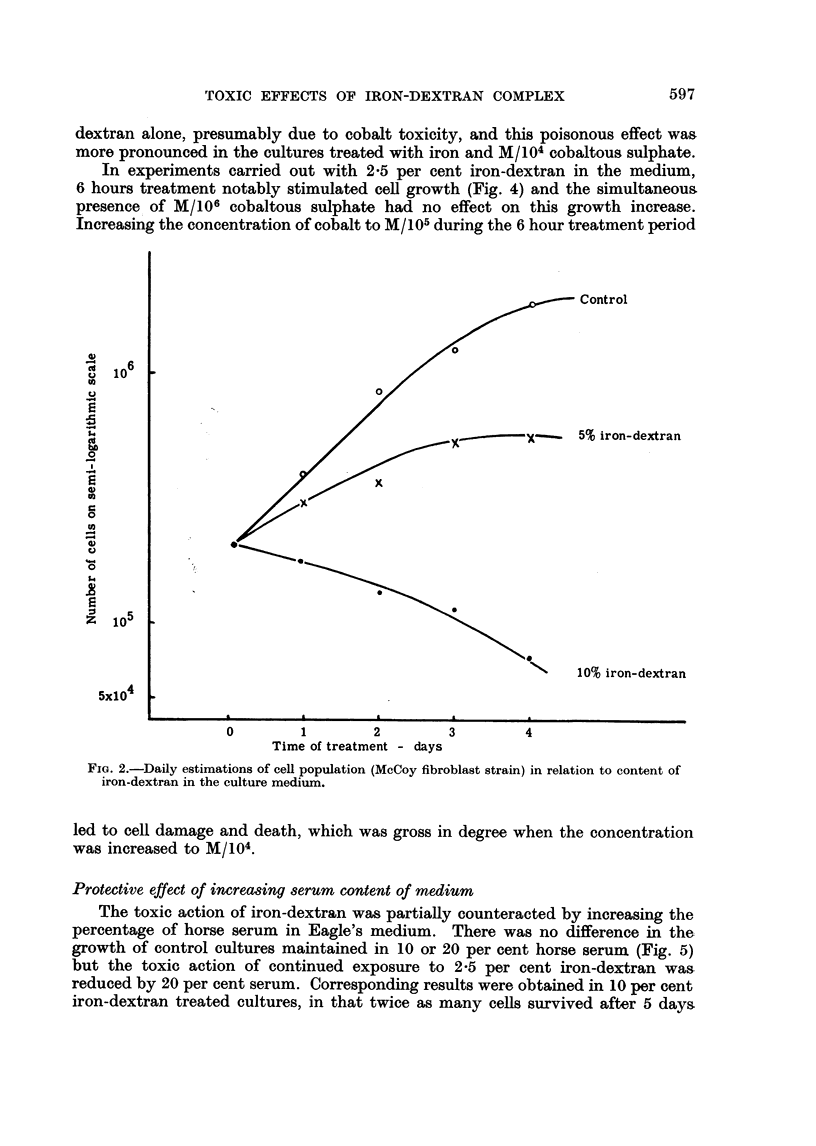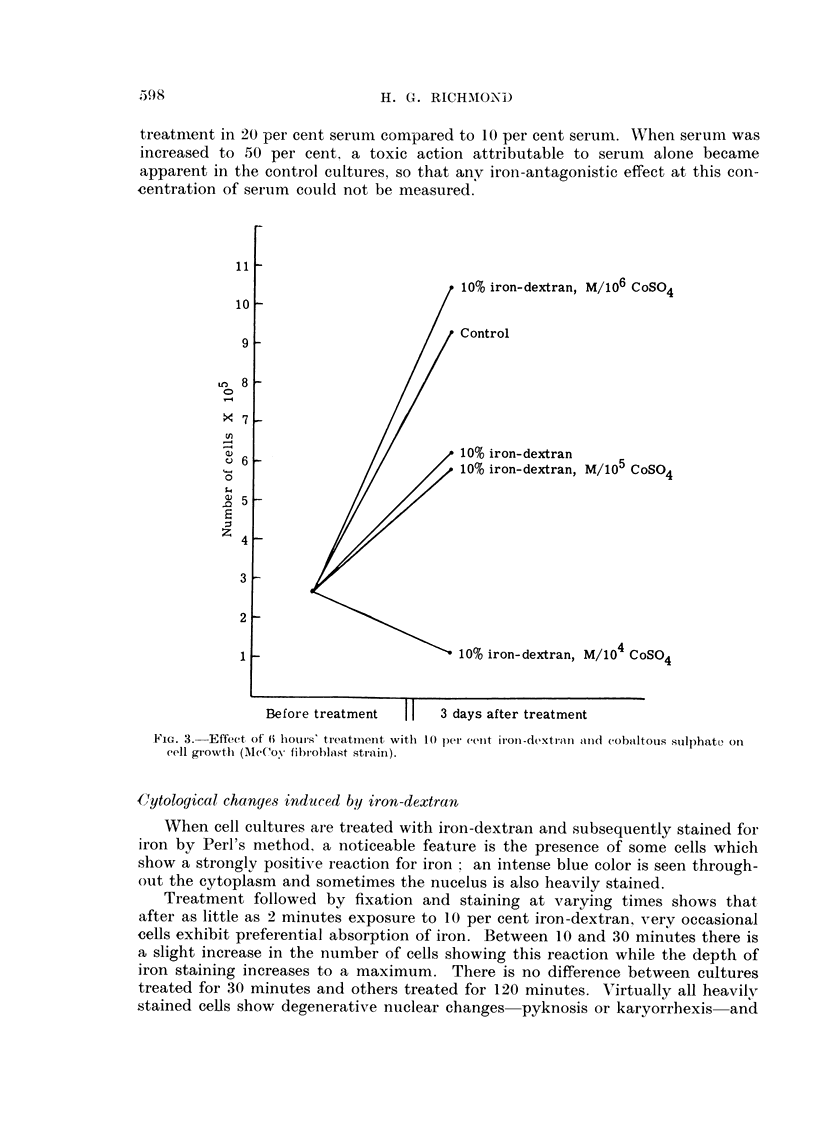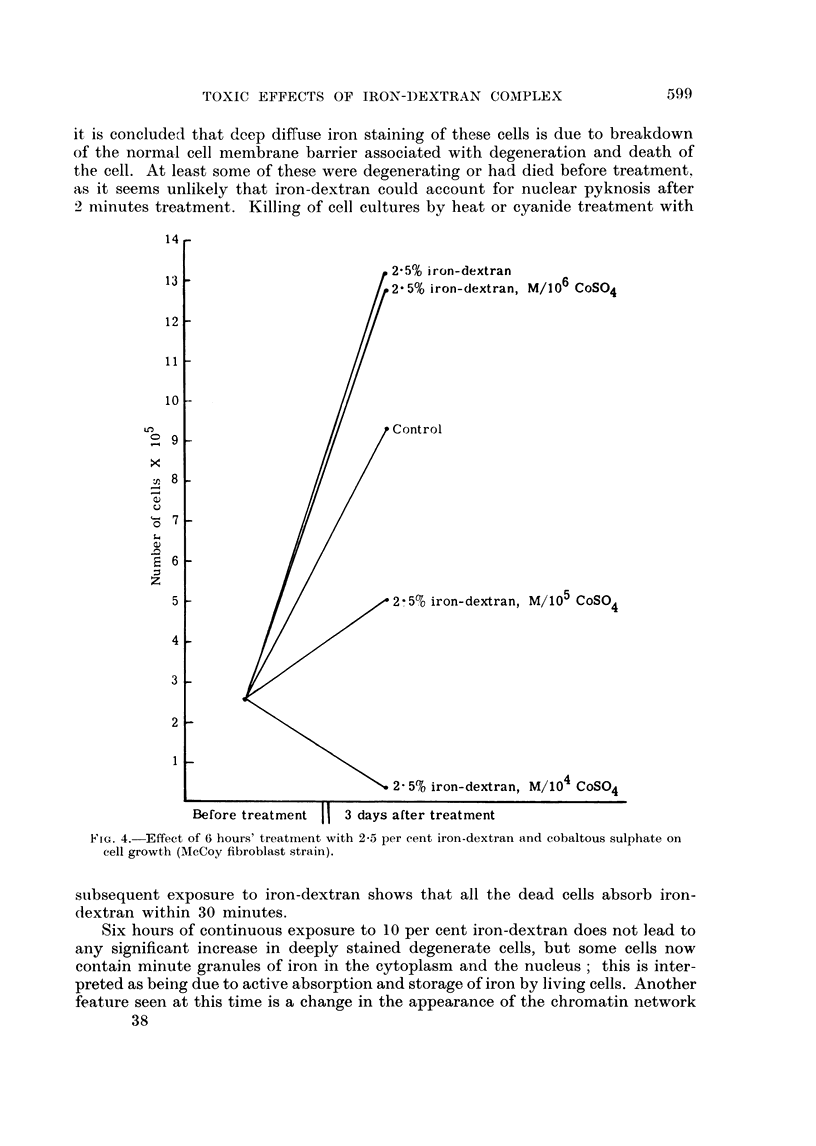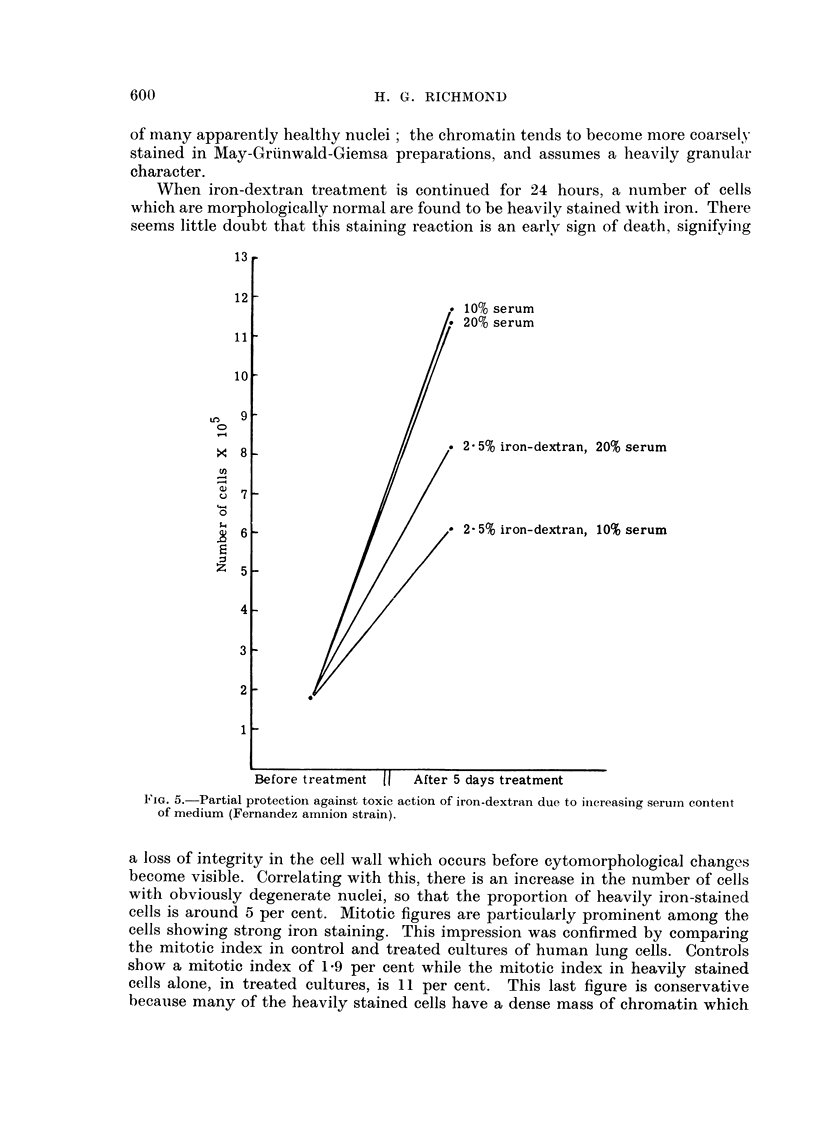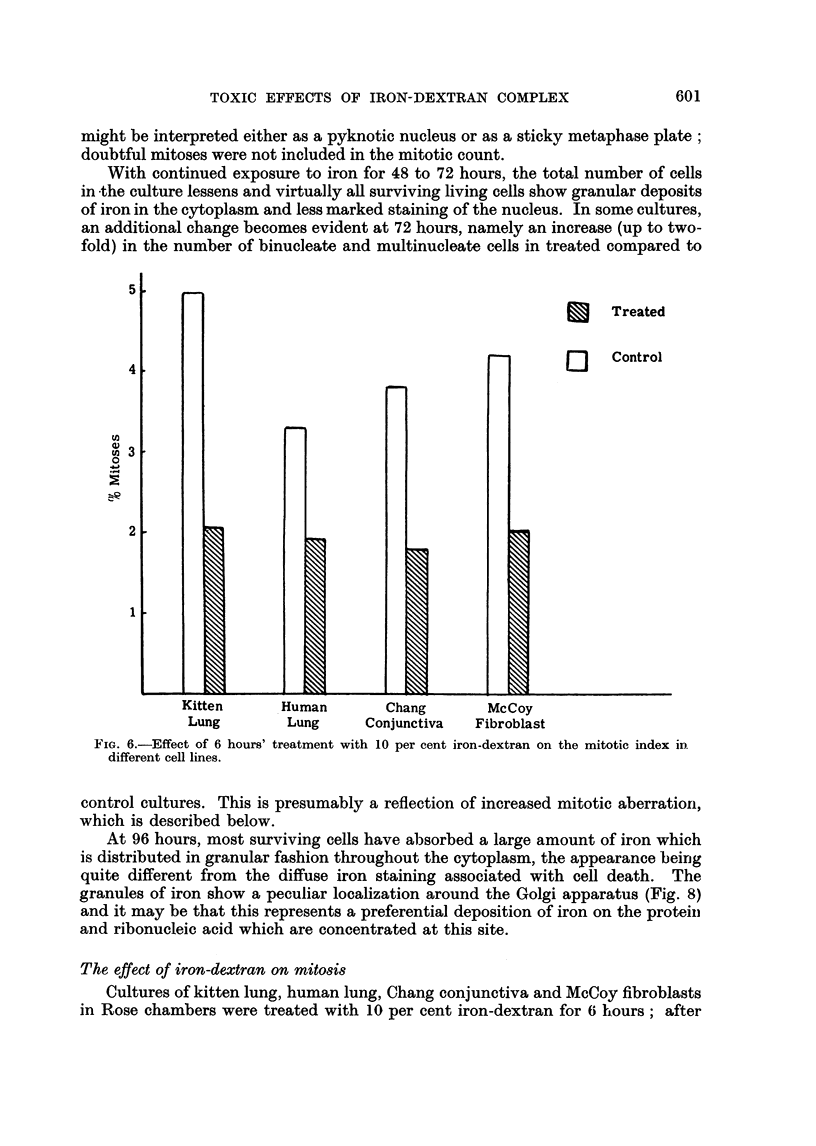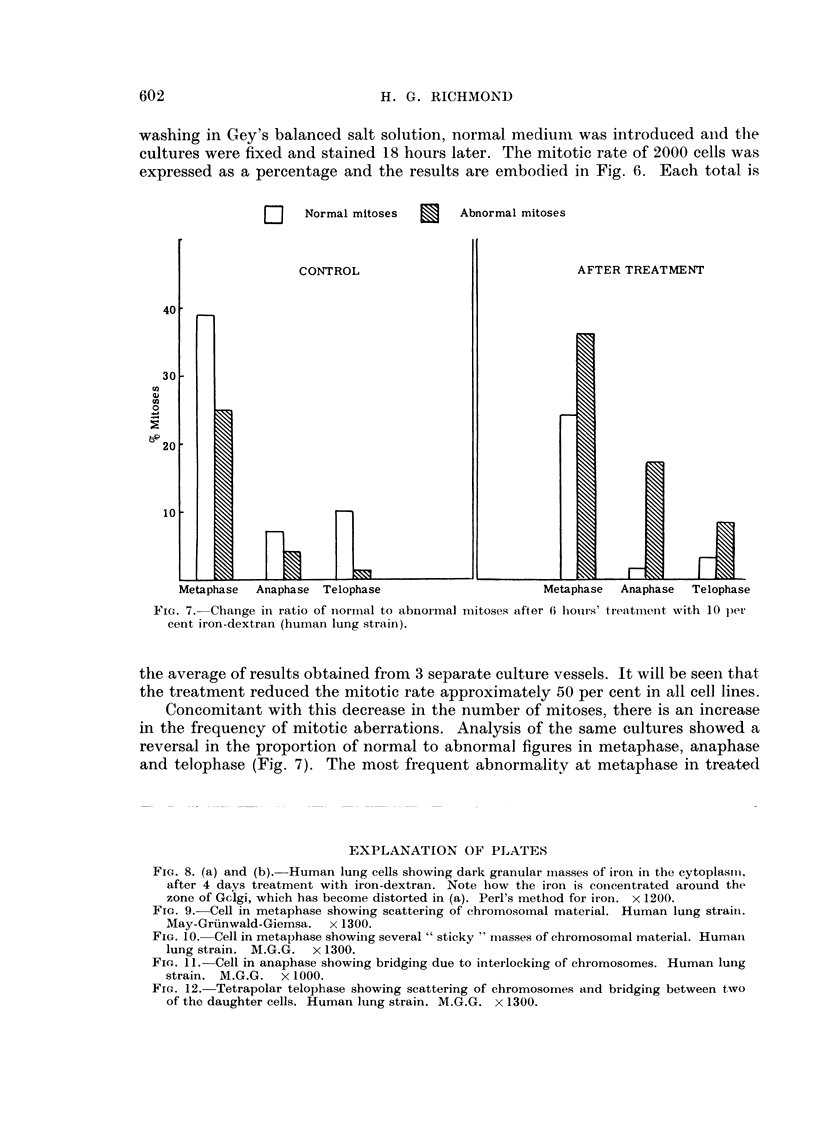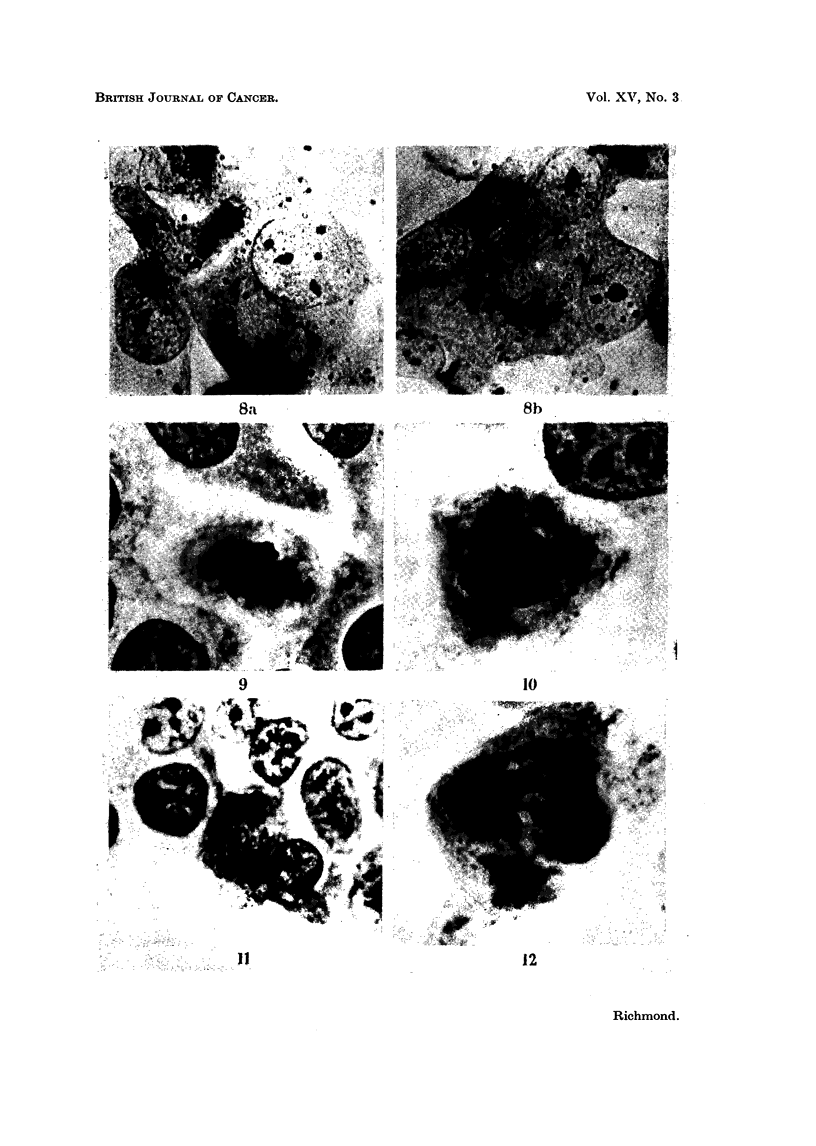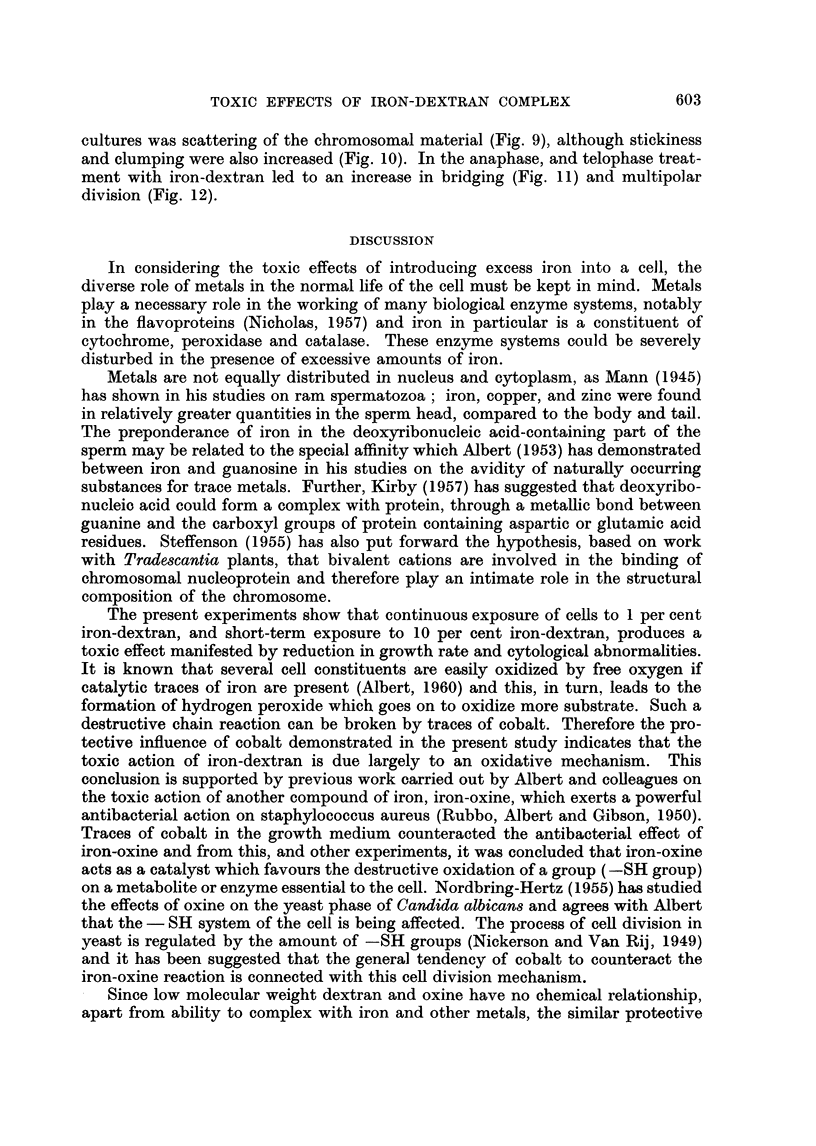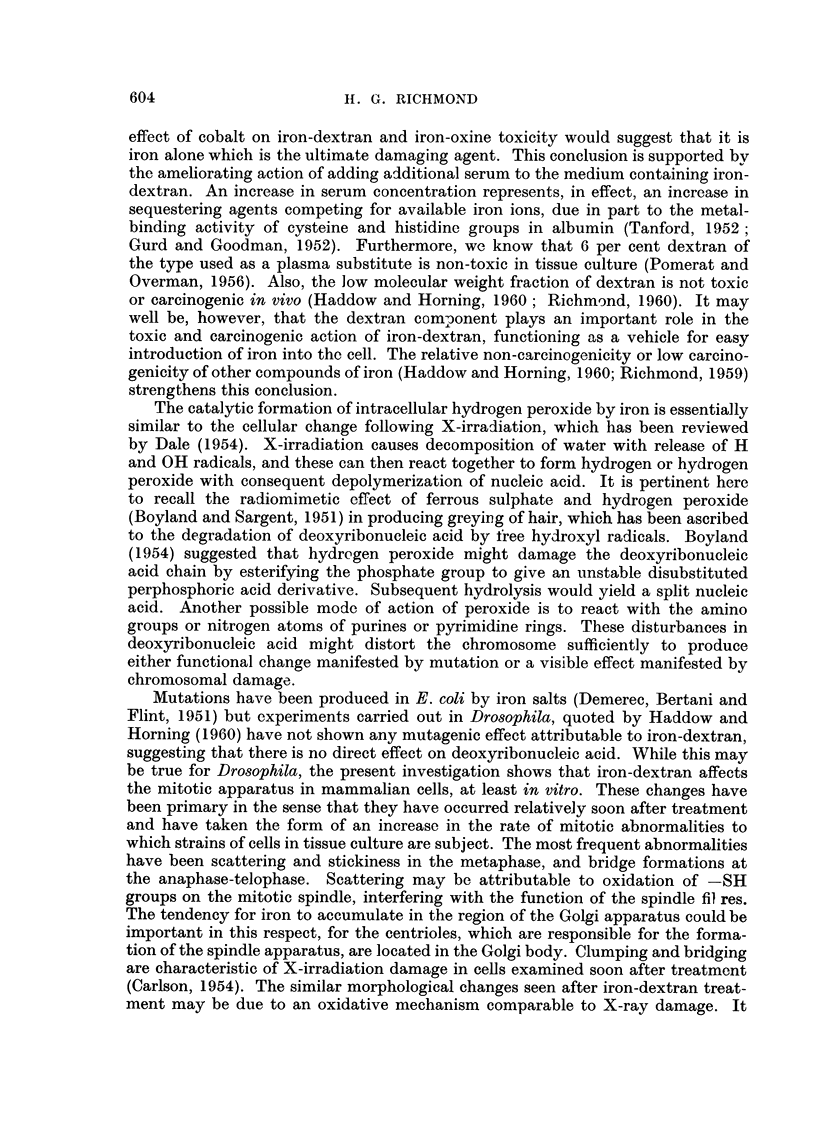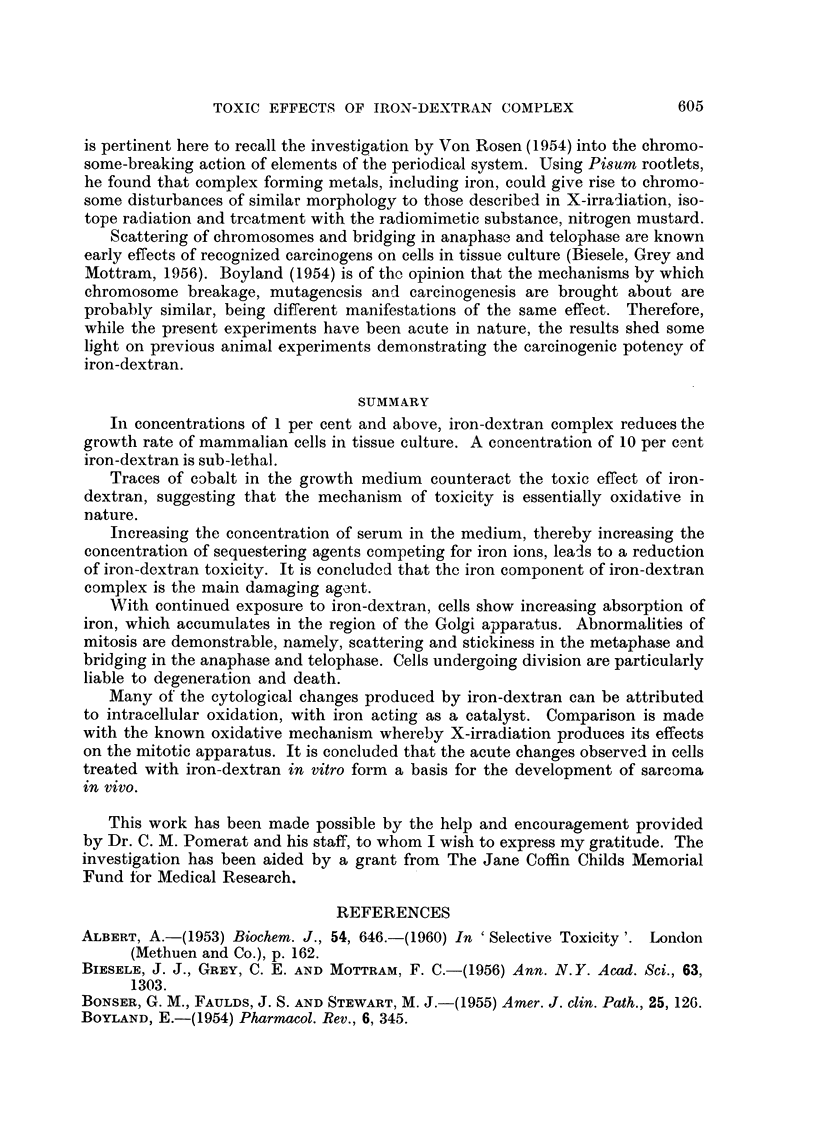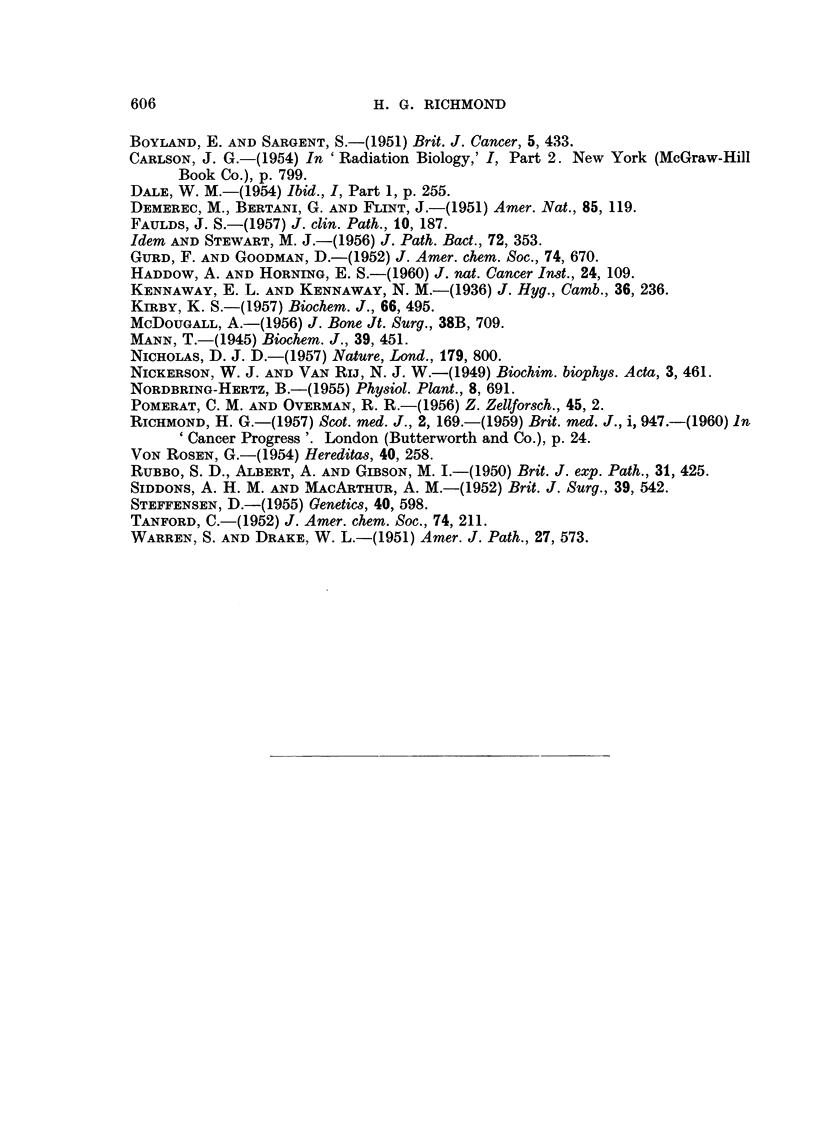# The Toxic Effects of Iron-Dextran Complex on Mammalian Cells in Tissue Culture

**DOI:** 10.1038/bjc.1961.69

**Published:** 1961-09

**Authors:** H. G. Richmond

## Abstract

**Images:**


### THE TOXIC EFFECTS OF IRON-DEXTRAN COMPLEX ON MAMMALIAN CELLS IN TISSUE CULTURE

H. G. RICHMOND*

*From the Pasadena Foundation for Medical Research, Pasadena, California, U.S.A.*†

Received for publication May 3, 1961

RECENT work (Richmond, 1957, 1959, 1960) has shown that iron-dextran complex is carcinogenic in the rat and mouse. These observations have been confirmed and extended by Haddow and Horning (1960). The dextran component of the complex is inactive, and whether the iron alone or the metal complex is the carcinogenic agent, the arresting problem of mechanism of action remains to be solved. While this question is of fundamental interest, there is a direct bearing on some aspects of human cancer since an association between lung cancer and metal in man has become clear in recent years. Kennaway and Kennaway reported in 1936 that lung cancer in England was over twice as common in metal grinders as in the general population. Haematite miners (Faulds and Stewart, 1956) and asbestos workers (Bonser, Faulds and Stewart, 1955) are also particularly prone to develop lung cancer. The similar occurrence of carcinoma of the lung in haematite miners, asbestos workers and chromate workers has been stressed by Faulds (1957) and since both asbestos and chromate contain varying proportions of iron in their chemical composition, it is possible that iron is the common carcinogenic factor. Attention may also be drawn to the high incidence of hepatoma in haemochromatosis (Warren and Drake, 1951) and the occurrence of carcinoma in relation to foreign metallic bodies (McDougall, 1956 ; Siddons and MacArthur, 1952).

With these facts in mind, it was considered desirable to investigate the effects of iron-dextran on cells grown *in vitro*, a system which allows fewer experimental variables than the intact animal. The present study concerns the acute reactions of growing cells *in vitro* on a short term basis. From previous experience *in vivo*, it was expected that a relatively high dose of iron-dextran would be necessary to produce immediate visible toxic damage, and this proved to be correct. It was hoped that elucidation of the mechanism of the immediate toxic reaction would afford some insight into the possible carcinogenic mechanism.

### MATERIALS

The several cell strains used in this investigation included human lung, kitten lung, Chang conjunctiva, Fernandez amnion and McCoy fibroblast. Cytological changes were observed by phase contrast microscopy in the living state and, after fixation, by bright field microscopy using preparations stained by May-Grünwald-Giemsa, Feulgen's reaction, crystal violet and Perl's method for iron.

Cultures were treated for varying periods of time by different concentrations of iron-dextran complex (Benger). This preparation consists of iron complexed

* Fellow of The Jane Coffin Childs Memorial Fund for Medical Research.
† Present address : Department of Pathology, Royal Northern Infirmary, Inverness, Scotland.

with a low molecular weight dextran fraction ; each 5 ml. contains the equivalent of 250 mg. iron calculated as Fe and present in the form of ferric hydroxide. The appropriate volume of iron-dextran was incorporated in the basic Eagle's medium with 10 per cent horse serum. Control cultures received simultaneous changes of normal medium ; in some experiments the control cultures were treated with normal medium containing a volume of sterile normal saline corresponding to the dose of iron-dextran.

In cell population studies, the Coulter electronic cell counter was used. The most suitable calibrations for counting were found to be a threshold of 20 and an aperture current setting of 4. As the error of the instrument count is in the range of 1 to 2 per cent, it did not appear necessary to correct for this error.

### EXPERIMENTAL

### *Effect of iron-dextran on growth of cell strains*

Equal numbers of cells, in the region of 200,000, were suspended in 5 ml. medium and implanted in T-30 flasks. After a period of 24 hours, during which the cells became firmly attached to the glass, the cells from 5 random flasks were harvested and counted in order to ensure that there had been equal distribution of cells to each flask. The remaining flasks were treated in groups of 5 with varying concentrations of iron-dextran except for a group of controls which received a change of normal medium. After a further 4 or 5 days at 37° C., the cells were harvested by trypsinization (1 per cent trypsin—Difco) for 3 minutes and their number was estimated, after suitable dilution in filtered saline, using the Coulter cell counter.

All strains showed a similar response to varying concentrations of iron-dextran, and the results in a kitten lung cell strain are depicted in Fig. 1 where each total represents the average content of 5 flasks. It will be seen that at a concentration of 0·5 per cent iron-dextran there is certainly no inhibition of growth, and there appears to be a slight stimulation. With increasing concentration of iron-dextran, growth is progressively inhibited and at the end of 4 days *in vitro* treatment, less than half the original number of cells implanted are now present in the flasks treated with 10 per cent iron-dextran. At concentrations of iron-dextran of 20 per cent and above, the cells were killed and " frozen " on the glass surface of the bottles.

Daily counts of population show a nearly logarithmic increase in the cell number in untreated control cultures, as seen in Fig. 2, where each point on the graph represents the average cell content of five T-30 flasks. Using 5 per cent iron-dextran in the medium, there is some inhibition of cell growth at 24 hours : after 48 hours the growth curve flattens and settles out into a plateau at a level which represents about one-half the potential growth in control cultures. The presence of 10 per cent iron-dextran in the medium leads to an immediate and continued fall in cell population.

### *Antagonistic effect of cobalt on toxic action of iron-dextran*

It was found that if cells were maintained continuously in a medium containing iron-dextran and cobalt, the cell numbers fell rapidly owing to the toxic action of cobalt. However, a protective effect due to the presence of cobalt could be demonstrated by the following technique.

Equal numbers of cells were implanted in T-30 flasks and, after 24 hours, groups of 5 flasks were treated for 6 hours by iron-dextran with and without cobaltous sulphate. Thereafter, the cultures were washed twice in Gey's balanced salt solution and fed with normal medium. Control cultures received corresponding changes of medium and cell populations of all flasks were estimated by the Coulter

Fig. 1.—Effect of varying concentrations of iron-dextran on cell populations (kitten lung strain) after 4 days growth in treated medium.

counter after 3 days growth following initial iron-dextran treatment. Under these conditions, the protective influence of cobalt became clear in the cells treated with 10 per cent iron-dextran (Fig. 3). Six hours' exposure to 10 per cent iron-dextran was moderately toxic to the cell cultures, but in the presence of iron-dextran and $M/10^6$ cobaltous sulphate, the toxic effect of iron was obviated and a mild stimulation of cell growth became evident. A combination of 10 per cent iron-dextran and $M/10^5$ cobaltous sulphate gave a reading similar to 10 per cent irondextran alone, presumably due to cobalt toxicity, and this poisonous effect was more pronounced in the cultures treated with iron and M/$10^4$ cobaltous sulphate.

In experiments carried out with 2·5 per cent iron-dextran in the medium, 6 hours treatment notably stimulated cell growth (Fig. 4) and the simultaneous presence of M/$10^6$ cobaltous sulphate had no effect on this growth increase. Increasing the concentration of cobalt to M/$10^5$ during the 6 hour treatment period

FIG. 2.—Daily estimations of cell population (McCoy fibroblast strain) in relation to content of iron-dextran in the culture medium.

led to cell damage and death, which was gross in degree when the concentration was increased to M/$10^4$.

*Protective effect of increasing serum content of medium*

The toxic action of iron-dextran was partially counteracted by increasing the percentage of horse serum in Eagle's medium. There was no difference in the growth of control cultures maintained in 10 or 20 per cent horse serum (Fig. 5) but the toxic action of continued exposure to 2·5 per cent iron-dextran was reduced by 20 per cent serum. Corresponding results were obtained in 10 per cent iron-dextran treated cultures, in that twice as many cells survived after 5 days treatment in 20 per cent serum compared to 10 per cent serum. When serum was increased to 50 per cent, a toxic action attributable to serum alone became apparent in the control cultures, so that any iron-antagonistic effect at this concentration of serum could not be measured.

FIG. 3.—Effect of 6 hours' treatment with 10 per cent iron-dextran and cobaltous sulphate on cell growth (McCoy fibroblast strain).

*Cytological changes induced by iron-dextran*

When cell cultures are treated with iron-dextran and subsequently stained for iron by Perl's method, a noticeable feature is the presence of some cells which show a strongly positive reaction for iron ; an intense blue color is seen throughout the cytoplasm and sometimes the nucelus is also heavily stained.

Treatment followed by fixation and staining at varying times shows that after as little as 2 minutes exposure to 10 per cent iron-dextran, very occasional cells exhibit preferential absorption of iron. Between 10 and 30 minutes there is a slight increase in the number of cells showing this reaction while the depth of iron staining increases to a maximum. There is no difference between cultures treated for 30 minutes and others treated for 120 minutes. Virtually all heavily stained cells show degenerative nuclear changes—pyknosis or karyorrhexis—and it is concluded that deep diffuse iron staining of these cells is due to breakdown of the normal cell membrane barrier associated with degeneration and death of the cell. At least some of these were degenerating or had died before treatment, as it seems unlikely that iron-dextran could account for nuclear pyknosis after 2 minutes treatment. Killing of cell cultures by heat or cyanide treatment with

FIG. 4.—Effect of 6 hours' treatment with 2·5 per cent iron-dextran and cobaltous sulphate on cell growth (McCoy fibroblast strain).

subsequent exposure to iron-dextran shows that all the dead cells absorb iron-dextran within 30 minutes.

Six hours of continuous exposure to 10 per cent iron-dextran does not lead to any significant increase in deeply stained degenerate cells, but some cells now contain minute granules of iron in the cytoplasm and the nucleus ; this is interpreted as being due to active absorption and storage of iron by living cells. Another feature seen at this time is a change in the appearance of the chromatin network of many apparently healthy nuclei ; the chromatin tends to become more coarsely stained in May-Grünwald-Giemsa preparations, and assumes a heavily granular character.

When iron-dextran treatment is continued for 24 hours, a number of cells which are morphologically normal are found to be heavily stained with iron. There seems little doubt that this staining reaction is an early sign of death, signifying

FIG. 5.—Partial protection against toxic action of iron-dextran due to increasing serum content of medium (Fernandez amnion strain).

a loss of integrity in the cell wall which occurs before cytomorphological changes become visible. Correlating with this, there is an increase in the number of cells with obviously degenerate nuclei, so that the proportion of heavily iron-stained cells is around 5 per cent. Mitotic figures are particularly prominent among the cells showing strong iron staining. This impression was confirmed by comparing the mitotic index in control and treated cultures of human lung cells. Controls show a mitotic index of 1·9 per cent while the mitotic index in heavily stained cells alone, in treated cultures, is 11 per cent. This last figure is conservative because many of the heavily stained cells have a dense mass of chromatin which might be interpreted either as a pyknotic nucleus or as a sticky metaphase plate ; doubtful mitoses were not included in the mitotic count.

With continued exposure to iron for 48 to 72 hours, the total number of cells in the culture lessens and virtually all surviving living cells show granular deposits of iron in the cytoplasm and less marked staining of the nucleus. In some cultures, an additional change becomes evident at 72 hours, namely an increase (up to two-fold) in the number of binucleate and multinucleate cells in treated compared to

FIG. 6.—Effect of 6 hours' treatment with 10 per cent iron-dextran on the mitotic index in different cell lines.

control cultures. This is presumably a reflection of increased mitotic aberration, which is described below.

At 96 hours, most surviving cells have absorbed a large amount of iron which is distributed in granular fashion throughout the cytoplasm, the appearance being quite different from the diffuse iron staining associated with cell death. The granules of iron show a peculiar localization around the Golgi apparatus (Fig. 8) and it may be that this represents a preferential deposition of iron on the protein and ribonucleic acid which are concentrated at this site.

*The effect of iron-dextran on mitosis*

Cultures of kitten lung, human lung, Chang conjunctiva and McCoy fibroblasts in Rose chambers were treated with 10 per cent iron-dextran for 6 hours ; after washing in Gey's balanced salt solution, normal medium was introduced and the cultures were fixed and stained 18 hours later. The mitotic rate of 2000 cells was expressed as a percentage and the results are embodied in Fig. 6. Each total is

FIG. 7.—Change in ratio of normal to abnormal mitoses after 6 hours' treatment with 10 per cent iron-dextran (human lung strain).

the average of results obtained from 3 separate culture vessels. It will be seen that the treatment reduced the mitotic rate approximately 50 per cent in all cell lines.

Concomitant with this decrease in the number of mitoses, there is an increase in the frequency of mitotic aberrations. Analysis of the same cultures showed a reversal in the proportion of normal to abnormal figures in metaphase, anaphase and telophase (Fig. 7). The most frequent abnormality at metaphase in treated

---

EXPLANATION OF PLATES

FIG. 8. (a) and (b).—Human lung cells showing dark granular masses of iron in the cytoplasm, after 4 days treatment with iron-dextran. Note how the iron is concentrated around the zone of Golgi, which has become distorted in (a). Perl's method for iron. ×1200.

FIG. 9.—Cell in metaphase showing scattering of chromosomal material. Human lung strain. May-Grünwald-Giemsa. ×1300.

FIG. 10.—Cell in metaphase showing several " sticky " masses of chromosomal material. Human lung strain. M.G.G. ×1300.

FIG. 11.—Cell in anaphase showing bridging due to interlocking of chromosomes. Human lung strain. M.G.G. ×1000.

FIG. 12.—Tetrapolar telophase showing scattering of chromosomes and bridging between two of the daughter cells. Human lung strain. M.G.G. ×1300.

8a 8b

9 10

11 12

Richmond.

cultures was scattering of the chromosomal material (Fig. 9), although stickiness and clumping were also increased (Fig. 10). In the anaphase, and telophase treatment with iron-dextran led to an increase in bridging (Fig. 11) and multipolar division (Fig. 12).

### DISCUSSION

In considering the toxic effects of introducing excess iron into a cell, the diverse role of metals in the normal life of the cell must be kept in mind. Metals play a necessary role in the working of many biological enzyme systems, notably in the flavoproteins (Nicholas, 1957) and iron in particular is a constituent of cytochrome, peroxidase and catalase. These enzyme systems could be severely disturbed in the presence of excessive amounts of iron.

Metals are not equally distributed in nucleus and cytoplasm, as Mann (1945) has shown in his studies on ram spermatozoa ; iron, copper, and zinc were found in relatively greater quantities in the sperm head, compared to the body and tail. The preponderance of iron in the deoxyribonucleic acid-containing part of the sperm may be related to the special affinity which Albert (1953) has demonstrated between iron and guanosine in his studies on the avidity of naturally occurring substances for trace metals. Further, Kirby (1957) has suggested that deoxyribonucleic acid could form a complex with protein, through a metallic bond between guanine and the carboxyl groups of protein containing aspartic or glutamic acid residues. Steffenson (1955) has also put forward the hypothesis, based on work with *Tradescantia* plants, that bivalent cations are involved in the binding of chromosomal nucleoprotein and therefore play an intimate role in the structural composition of the chromosome.

The present experiments show that continuous exposure of cells to 1 per cent iron-dextran, and short-term exposure to 10 per cent iron-dextran, produces a toxic effect manifested by reduction in growth rate and cytological abnormalities. It is known that several cell constituents are easily oxidized by free oxygen if catalytic traces of iron are present (Albert, 1960) and this, in turn, leads to the formation of hydrogen peroxide which goes on to oxidize more substrate. Such a destructive chain reaction can be broken by traces of cobalt. Therefore the protective influence of cobalt demonstrated in the present study indicates that the toxic action of iron-dextran is due largely to an oxidative mechanism. This conclusion is supported by previous work carried out by Albert and colleagues on the toxic action of another compound of iron, iron-oxine, which exerts a powerful antibacterial action on staphylococcus aureus (Rubbo, Albert and Gibson, 1950). Traces of cobalt in the growth medium counteracted the antibacterial effect of iron-oxine and from this, and other experiments, it was concluded that iron-oxine acts as a catalyst which favours the destructive oxidation of a group (—SH group) on a metabolite or enzyme essential to the cell. Nordbring-Hertz (1955) has studied the effects of oxine on the yeast phase of *Candida albicans* and agrees with Albert that the — SH system of the cell is being affected. The process of cell division in yeast is regulated by the amount of —SH groups (Nickerson and Van Rij, 1949) and it has been suggested that the general tendency of cobalt to counteract the iron-oxine reaction is connected with this cell division mechanism.

Since low molecular weight dextran and oxine have no chemical relationship, apart from ability to complex with iron and other metals, the similar protective effect of cobalt on iron-dextran and iron-oxine toxicity would suggest that it is iron alone which is the ultimate damaging agent. This conclusion is supported by the ameliorating action of adding additional serum to the medium containing iron-dextran. An increase in serum concentration represents, in effect, an increase in sequestering agents competing for available iron ions, due in part to the metal-binding activity of cysteine and histidine groups in albumin (Tanford, 1952 ; Gurd and Goodman, 1952). Furthermore, we know that 6 per cent dextran of the type used as a plasma substitute is non-toxic in tissue culture (Pomerat and Overman, 1956). Also, the low molecular weight fraction of dextran is not toxic or carcinogenic *in vivo* (Haddow and Horning, 1960 ; Richmond, 1960). It may well be, however, that the dextran component plays an important role in the toxic and carcinogenic action of iron-dextran, functioning as a vehicle for easy introduction of iron into the cell. The relative non-carcinogenicity or low carcinogenicity of other compounds of iron (Haddow and Horning, 1960; Richmond, 1959) strengthens this conclusion.

The catalytic formation of intracellular hydrogen peroxide by iron is essentially similar to the cellular change following X-irradiation, which has been reviewed by Dale (1954). X-irradiation causes decomposition of water with release of H and OH radicals, and these can then react together to form hydrogen or hydrogen peroxide with consequent depolymerization of nucleic acid. It is pertinent here to recall the radiomimetic effect of ferrous sulphate and hydrogen peroxide (Boyland and Sargent, 1951) in producing greying of hair, which has been ascribed to the degradation of deoxyribonucleic acid by free hydroxyl radicals. Boyland (1954) suggested that hydrogen peroxide might damage the deoxyribonucleic acid chain by esterifying the phosphate group to give an unstable disubstituted perphosphoric acid derivative. Subsequent hydrolysis would yield a split nucleic acid. Another possible mode of action of peroxide is to react with the amino groups or nitrogen atoms of purines or pyrimidine rings. These disturbances in deoxyribonucleic acid might distort the chromosome sufficiently to produce either functional change manifested by mutation or a visible effect manifested by chromosomal damage.

Mutations have been produced in *E. coli* by iron salts (Demerec, Bertani and Flint, 1951) but experiments carried out in *Drosophila*, quoted by Haddow and Horning (1960) have not shown any mutagenic effect attributable to iron-dextran, suggesting that there is no direct effect on deoxyribonucleic acid. While this may be true for *Drosophila*, the present investigation shows that iron-dextran affects the mitotic apparatus in mammalian cells, at least *in vitro*. These changes have been primary in the sense that they have occurred relatively soon after treatment and have taken the form of an increase in the rate of mitotic abnormalities to which strains of cells in tissue culture are subject. The most frequent abnormalities have been scattering and stickiness in the metaphase, and bridge formations at the anaphase-telophase. Scattering may be attributable to oxidation of —SH groups on the mitotic spindle, interfering with the function of the spindle fil res. The tendency for iron to accumulate in the region of the Golgi apparatus could be important in this respect, for the centrioles, which are responsible for the formation of the spindle apparatus, are located in the Golgi body. Clumping and bridging are characteristic of X-irradiation damage in cells examined soon after treatment (Carlson, 1954). The similar morphological changes seen after iron-dextran treatment may be due to an oxidative mechanism comparable to X-ray damage. It is pertinent here to recall the investigation by Von Rosen (1954) into the chromosome-breaking action of elements of the periodical system. Using *Pisum* rootlets, he found that complex forming metals, including iron, could give rise to chromosome disturbances of similar morphology to those described in X-irradiation, isotope radiation and treatment with the radiomimetic substance, nitrogen mustard.

Scattering of chromosomes and bridging in anaphase and telophase are known early effects of recognized carcinogens on cells in tissue culture (Biesele, Grey and Mottram, 1956). Boyland (1954) is of the opinion that the mechanisms by which chromosome breakage, mutagenesis and carcinogenesis are brought about are probably similar, being different manifestations of the same effect. Therefore, while the present experiments have been acute in nature, the results shed some light on previous animal experiments demonstrating the carcinogenic potency of iron-dextran.

### SUMMARY

In concentrations of 1 per cent and above, iron-dextran complex reduces the growth rate of mammalian cells in tissue culture. A concentration of 10 per cent iron-dextran is sub-lethal.

Traces of cobalt in the growth medium counteract the toxic effect of iron-dextran, suggesting that the mechanism of toxicity is essentially oxidative in nature.

Increasing the concentration of serum in the medium, thereby increasing the concentration of sequestering agents competing for iron ions, leads to a reduction of iron-dextran toxicity. It is concluded that the iron component of iron-dextran complex is the main damaging agent.

With continued exposure to iron-dextran, cells show increasing absorption of iron, which accumulates in the region of the Golgi apparatus. Abnormalities of mitosis are demonstrable, namely, scattering and stickiness in the metaphase and bridging in the anaphase and telophase. Cells undergoing division are particularly liable to degeneration and death.

Many of the cytological changes produced by iron-dextran can be attributed to intracellular oxidation, with iron acting as a catalyst. Comparison is made with the known oxidative mechanism whereby X-irradiation produces its effects on the mitotic apparatus. It is concluded that the acute changes observed in cells treated with iron-dextran *in vitro* form a basis for the development of sarcoma *in vivo*.

This work has been made possible by the help and encouragement provided by Dr. C. M. Pomerat and his staff, to whom I wish to express my gratitude. The investigation has been aided by a grant from The Jane Coffin Childs Memorial Fund for Medical Research.

### REFERENCES

ALBERT, A.—(1953) *Biochem. J.*, **54**, 646.—(1960) *In* ' Selective Toxicity '. London (Methuen and Co.), p. 162.

BIESELE, J. J., GREY, C. E. AND MOTTRAM, F. C.—(1956) *Ann. N.Y. Acad. Sci.*, **63**, 1303.

BONSER, G. M., FAULDS, J. S. AND STEWART, M. J.—(1955) *Amer. J. clin. Path.*, **25**, 126.

BOYLAND, E.—(1954) *Pharmacol. Rev.*, **6**, 345.

Boyland, E. and Sargent, S.—(1951) *Brit. J. Cancer*, **5**, 433.
Carlson, J. G.—(1954) *In* ' Radiation Biology,' *I*, Part 2. New York (McGraw-Hill Book Co.), p. 799.
Dale, W. M.—(1954) *Ibid.*, *I*, Part 1, p. 255.
Demerec, M., Bertani, G. and Flint, J.—(1951) *Amer. Nat.*, **85**, 119.
Faulds, J. S.—(1957) *J. clin. Path.*, **10**, 187.
*Idem* and Stewart, M. J.—(1956) *J. Path. Bact.*, **72**, 353.
Gurd, F. and Goodman, D.—(1952) *J. Amer. chem. Soc.*, **74**, 670.
Haddow, A. and Horning, E. S.—(1960) *J. nat. Cancer Inst.*, **24**, 109.
Kennaway, E. L. and Kennaway, N. M.—(1936) *J. Hyg., Camb.*, **36**, 236.
Kirby, K. S.—(1957) *Biochem. J.*, **66**, 495.
McDougall, A.—(1956) *J. Bone Jt. Surg.*, **38**B, 709.
Mann, T.—(1945) *Biochem. J.*, **39**, 451.
Nicholas, D. J. D.—(1957) *Nature, Lond.*, **179**, 800.
Nickerson, W. J. and Van Rij, N. J. W.—(1949) *Biochim. biophys. Acta*, **3**, 461.
Nordbring-Hertz, B.—(1955) *Physiol. Plant.*, **8**, 691.
Pomerat, C. M. and Overman, R. R.—(1956) *Z. Zellforsch.*, **45**, 2.
Richmond, H. G.—(1957) *Scot. med. J.*, **2**, 169.—(1959) *Brit. med. J.*, i, 947.—(1960) *In* ' Cancer Progress '. London (Butterworth and Co.), p. 24.
Von Rosen, G.—(1954) *Hereditas*, **40**, 258.
Rubbo, S. D., Albert, A. and Gibson, M. I.—(1950) *Brit. J. exp. Path.*, **31**, 425.
Siddons, A. H. M. and MacArthur, A. M.—(1952) *Brit. J. Surg.*, **39**, 542.
Steffensen, D.—(1955) *Genetics*, **40**, 598.
Tanford, C.—(1952) *J. Amer. chem. Soc.*, **74**, 211.
Warren, S. and Drake, W. L.—(1951) *Amer. J. Path.*, **27**, 573.